# Preferential effects of low volume versus high volume replacement with crystalloid fluid in a hemorrhagic shock model in pigs

**DOI:** 10.1186/s12871-015-0114-9

**Published:** 2015-10-06

**Authors:** Martin Ponschab, Herbert Schöchl, Claudia Keibl, Henrik Fischer, Heinz Redl, Christoph J. Schlimp

**Affiliations:** Ludwig Boltzmann Institute for Experimental and Clinical Traumatology, AUVA Research Centre, Donaueschingenstrasse 13, A-1200, Vienna Austria; Department of Anaesthesiology and Intensive Care, AUVA Trauma Hospital, Linz, Austria; Department of Anaesthesiology and Intensive Care, AUVA Trauma Centre, Salzburg, Austria; Department I/10, Federal Ministry of the Interior, Vienna, Republic of Austria; Department of Anaesthesiology and Intensive Care, AUVA Trauma Hospital, Klagenfurt, Austria

**Keywords:** Crystalloid fluid, Fluid resuscitation, Haemorrhage, Thromboelastometry, Experimental pig model

## Abstract

**Background:**

Fluid resuscitation is a core stone of hemorrhagic shock therapy, and crystalloid fluids seem to be associated with lower mortality compared to colloids. However, as redistribution starts within minutes, it has been suggested to replace blood loss with a minimum of a three-fold amount of crystalloids. The hypothesis was that in comparison to high volume (HV), a lower crystalloid volume (LV) achieves a favorable coagulation profile and exerts sufficient haemodynamics in the acute phase of resuscitation.

**Methods:**

In 24 anaesthetized pigs, controlled arterial blood loss of 50 % of the estimated blood volume was either (*n* = 12) replaced with a LV (one-fold) or a HV (three-fold) volume of a balanced, acetated crystalloid solution at room temperature. Hemodynamic parameters, dilution effects and coagulation profile by standard coagulation tests and thromboelastometry at baseline and after resuscitation were determined in both groups.

**Results:**

LV resuscitation increased MAP significantly less compared to the HV, 61 ± 7 vs. 82 ± 14 mmHg (*p* < 0.001) respectively, with no difference between lactate and base excess between groups. Haematocrit after fluid replacement was 0.20 vs. 0.16 (LV vs. HV, *p* < 0.001), suggesting a grade of blood dilution of 32 vs. 42 % (*p* < 0.001) compared to baseline values. Compared to LV, HV resulted in decreased core temperature (37.5 ± 0.2 vs. 36.0 ± 0.6 °C, *p* < 0.001), lower platelet count (318 ± 77 vs. 231 ± 53 K/μL, *p* < 0.01) and lower plasma fibrinogen levels (205 ± 19 vs. 168 ± 24 mg/dL, *p* < 0.001). Thromboelastometric measurements showed a significant impairment on viscoelastic clot properties following HV group. While prothrombin time index decreased significantly more in the HV group, activated partial thromboplastin time did not differ between both groups. HV did not result in hyperchloraemic acidosis.

**Discussion:**

Coagulation parameters represented by plasma fibrinogen and ROTEM parameters were also less impaired with LV. With regrad to hematocrit, 60 % of LV remained intracascular , while in HV only 30 % remained in circulation within the first hour of administration. In the acute setting of 50 % controlled blood loss, a one fold LV crystalloid replacement strategy is sufficient to adequately raise blood pressure up to a mean arterial pressure >50 mm Hg. The concept of damage control resuscitation (DCR) with permissive hypotension may be better met by using LV as compared to a three fold HV resuscitation strategy.

**Conclusion:**

High volume administration of an acetated balanced crystalloid does not lead to hyperchloraemic acidosis, but may negatively influence clinical parameters, such as higher blood pressure, lower body temperature and impaired coagulation parameters, which could potentially increase bleeding after trauma. Replacement of acute blood loss with just an equal amount of an acetated balanced crystalloid appears to be the preferential treatment strategy in the acute phase after controlled bleeding.

## Background

In order to ensure tissue oxygenation it seems to be essential to restore the circulating blood volume. Thus, fluid resuscitation is mandatory in hemorrhagic shock therapy to maintain adequate blood flow and blood pressure [1]. Nevertheless, it has been questioned whether normalization of blood pressure in bleeding patients is harmful [2]. Both crystalloids and colloids are widely used for initial fluid therapy. However the use of artificial colloids for volume replacement is still under debate [3]. A metaanalysis found that crystalloids are associated with lower mortality in trauma patients compared to colloids [4].

Current European guidelines on the management of severe perioperative bleeding [5] and bleeding management in trauma [6] recommend replacement of extracellular fluid losses with isotonic crystalloids in a timely and protocol-based manner. In the setting of acute bleeding bolus infusion up to two liters of crystalloids in an adult (70 kg) patient is recommended as initial fluid therapy [7, 8].

When using large quantities of crystalloid solutions such as normal saline, development of dilutional hyperchloraemic acidosis has been described [9]. It is well known that acidosis impairs coagulation, particularly platelet function and thrombin generation [10, 11]. Therefore, it has been speculated that balanced solutions may potentially avoid or minimize these effects, and current European guidelines on the management of severe perioperative bleeding recommend the use of balanced solutions for crystalloids [5]. In balanced crystalloids, metabolic anions (mainly acetate or lactate) are used instead of chloride to establish neutrality of electrons and isotonicity in vitro. From a physiological point of view, the intravascular volume effect of crystalloids can be less than 20 % and redistribution starts within minutes [12]. Therefore it has been suggested to replace blood loss with at least a three-fold amount of crystalloids or more [13–15].

We aimed to investigate fluid replacement with a low (one-fold, LV) or a high (three-fold, HV) volume of a modern, balanced, acetated crystalloid solution in a pig model of controlled arterial blood loss of 50 % of the estimated blood volume. The effects on basic haemodynamic monitoring, acid base status, electrolyte status, standard coagulation tests and thromboelastometric parameters were investigated.

We decided to use this model of controlled haemorrhage with approximately 50 % blood loss within 30 min as the average transport time to hospital in traumatological emergency systems in middle Europe is comparable [16]. Our haemorrhagic shock model is comparable to other shock models in pigs, where shed blood volume amounted to 40–50 %, or shock was achieved by 30 ml/kg withdrawl of blood, and MAP below 35 mmHg [17–19].

We hypothesized that LV fluid replacement would result in suitable blood pressure improvement (mean arterial pressure >50 mmHg), similar acid base balance and lactate production, but less impairment of coagulation as compared to HV fluid therapy.

## Methods

### Animals

The experimental protocol was approved by the Animal Protocol Review Board of the City Government of Vienna, Austria under protocol number MA58-005750/2012/9, and our centre is certified by the same Review Board for performing animal studies. All experiments were performed under conditions described in the *Guide for the Care and Use of Laboratory Animals*, as defined by the National Institutes of Health.

Twentyfour healthy male pigs (Landrace pigs from a government-approved farmer in Münichsthal, Austria; age range 12–16 weeks) were used for the investigation. The blood volume of the actual pigs was estimated with 70 ml/kg.

Two days prior to the study, the animals were housed in pairs and on straw, with unrestricted access to food (farmers’ domestically produced diet) and water. Temperature was maintained between 19 and 23 °C, relative humidity was 55 ± 10 %, and a 12/12 h light/dark cycle was maintained. Animals were fasted overnight before surgical procedures, with unrestricted access to water.

### Anaesthesia, surgical preparations and cardiorespiratory monitoring

Anaesthesia was induced intramuscularly with a combination of butorphanol (0.17 mg/kg; Alvetra and Werfft AG, Vienna, Austria), medetomidine (0.03 mg/kg; Eurovet Animal Health, Bladel, Netherlands) and midazolam (0.5 mg/kg; Nycomed Austria GmbH, Linz, Austria) followed by intravenous (ear vein) ketamine (7 mg/kg; Pfizer, Vienna, Austria). Intubation was performed with a 6.5 mm tracheal tube, and volume controlled ventilation was set to maintain end-tidal CO_2_ between 4.5 and 5.5 kPa. Anaesthesia was maintained intravenously with midazolam (0.8 mg/kg/h), sufentanil (8 μg/kg/h; Janssen, Vienna, Austria) and rocuronium (5 mg/kg/h; Organon, Oss, Netherlands). Catheters were placed by direct preparation and incision of the left external jugular vein and left carotid artery for standard fluid therapy, invasive blood pressure monitoring and blood withdrawal (according to the standardized pig anaesthesia protocol). The arterial catheter was constantly flushed at a rate of 4 ml/h using heparinised saline (8 U/mL heparine) to prevent clotting. A 14-Fr catheter was inserted suprapubically into the bladder. Mean arterial pressure, heart rate, oxygen saturation and ventilation parameters were monitored continuously, while arterial blood gas analysis and lactate were measured at blood sampling time-points. Experiments were performed at the in-house operation theatre in the morning. Safety of interventions was assessed by haemodynamic monitoring (blood pressure, heart rate) throughout the whole experiment.

### Experimental protocol

**Fig. 1:**
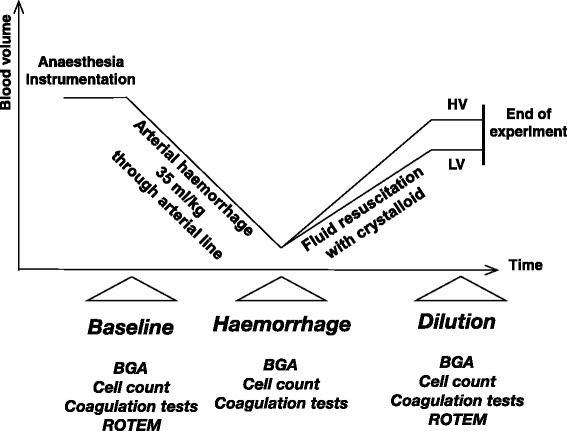
Schematic description of the experimental procedure, time-points and laboratory investigation of the used pig model with 50 % controlled haemorrhage followed by two different fluid replacement regimen using an acetated, balanced crystalloid either with a 1-fold, low (LV, *n* = 12) or a 3-fold, high (HV, *n* = 12) volume

A schematic illustration of the experimental protocol is depicted in Fig. 1. Pigs selected for instrumentation by the animal care keepers of the animal research laboratory were randomly assigned to the low or high volume group according to a written study plan.

After induction of anaesthesia and instrumentation, the first study measurements (baseline) were carried out. A substantial bleeding situation was then simulated by withdrawing 35 ml/kg blood through an arterial catheter within a period of 30 min (withdrawal rate approximately 30–50 ml/min). ELO-MEL® (Fresenius Kabi, Wien, Austria) a modern, balanced and acetated crystalloid, which contains sodium (140.0 mmol/L), potassium (5.0 mmol/L), calcium (2.5 mmol/L), magnesium (1.5 mmol/L), chloride (108.0 mmol/L) and acetate (45.0 mmol/L) has been used for fluid replacement therapy. The osmolality is reported to be 302 mosmol/L, and the pH value of the solution ranges between 6.0 and 7.5. Directly after completion of measurement after blood loss, fluid administration of either 1000 ml (LV) or 3000 ml (HV) of a balanced crystalloid solution (ELO-MEL isoton, Fresenius Kabi, Graz, Austria) was administered within a time of approximately 40 min. Ten minutes after cessation of fluid infusion measurements were completed.

### Study measurements, blood sampling and analytical methods

Baseline arterial blood samples were measured at the beginning of the experiment. For blood gas analysis, blood was collected in a 1 ml heparinized syringe and analysis measurement of electrolytes, lactate and glucose was carried out on an ABL 870 Flex (Radiometer, Copenhagen, Denmark). For measurement of haematocrit (Hct)), white blood cell count (WBC) and platelet count (Plt) blood was collected in 3-mL tri-potassium ethylenediaminetetraacetic acid (K_3_EDTA) tubes (Vacuette), with the addition of 1.6 mg/mL EDTA. Cell counts were measured with a CELLDYN 3700 Instrument (Abbott, Vienna, Austria), using appropriate animal settings. The ratio of Hct after fluid replacement to Hct at baseline was used to estimate a percentage of dilution. For the coagulation analyses, blood was initially collected in 3.5 mL tubes containing 0.35 mL buffered 3.2 % trisodium citrate (Vacuette; Greiner Bio-One, Linz, Austria), giving a volume ratio for citrate to whole blood of 1:9. Aliquots of citrated blood were centrifuged immediately at 2800 g for 15 min to obtain plasma. Fibrinogen (Clauss method; Multifibren U, Siemens, Marburg, Germany) and activated partial thromboplastin time (aPTT) (Actin FS; Siemens, Marburg, Germany) were analysed with a KC-10 steel ball coagulometer (Amelung GmbH, Lemco, Germany). Prothrombin time index (PTI) (Thromborel S; Siemens, Marburg, Germany) was run on a Sysmex CA 1500 device (Siemens, Marburg, Germany).

### Thromboelastometric measurements

Thromboelastometric measurements were carried out at baseline and after fluid replacement only. The ROTEM device was checked for correct functioning according to the manufacturer’s recommendation by using control plasma (ROTROL N). ROTEM analyses were then performed in two ways: 1) standard EXTEM and FIBTEM assays immediately after citrated whole blood was drawn; and 2) EXTEM assay in platelet-free plasma (PFP), obtained by centrifuging (2800 g for 15 min) and filtering (0.22 μm) the citrated blood samples.

All assays were initiated by the addition of 20 μL CaCl_2_, 200 mmol/L and 20 μL Ex-Tem® reagent (Tem International GmbH, Munich, Germany), providing extrinsic activation through tissue factor. In the FIBTEM assay, additional cytochalasin D inhibits platelets’ contribution to clot strength by preventing cytoskeletal reorganisation. Because the commercially available FIBTEM assay does not sufficiently inhibit platelets in porcine blood samples, we also performed the EXTEM in PFP to estimate fibrin polymerization in plasma without platelets [20]. The following ROTEM variables were analysed: clotting time (CT [sec]; time from the start of measurement until formation of a clot of 2 mm in amplitude); clot formation time (CFT [sec]; time from the end of CT until a clot firmness of 20 mm was achieved); and maximum clot firmness (MCF [mm]; maximum strength of the clot, determined by the interaction of fibrin, activated platelets and factor XIII). In FIBTEM and PFP EXTEM, only MCF was analysed.

### Statistical analysis

The number of animals (*n* = 12 per group) included in the study was based on pilot experiments and a consecutive power analysis, considering a drop out of 20 % and assuming that HV fluid replacement (as compared to LV) may reduce plasma fibrinogen (the most sensible coagulation factor to decline after haemorrhage and dilution) [21] by 50 ± 25 mg/dL and providing at a power of 90 % and a two-sided alpha of 5 %. Normal distribution of data was evaluated using the Kolmogorov-Smirnov test. Normally distributed data were expressed as mean ± standard deviation, and data not following the normal distribution were expressed as median and interquartile range. A repeated measures analysis of variance (ANOVA) was used to detect differences between time-points (baseline, haemorrhage, dilution) and a Tukey’s *post hoc* correction for multiple comparisons was applied. The Student’s *t*-test (normal distribution) or the Mann–Whitney test (non-normal distribution) was used to assess differences between the two groups at each time-point. A two-tailed *P*-value <0.05 was considered statistically significant. All statistical calculations were performed using commercially available statistical software (GraphPad Prism 5, GraphPad Software, La Jolla, CA).

## Results

All animals were treated according to the experimental protocol (Fig. 1). The mean body weight was 33.9 ± 3.4 kg (range 27.5–40.5 kg), mean blood loss was 1187 ± 120 ml and the time between blood sampling at baseline and after haemorrhage was 45 ± 8 min in LV and 41 ± 7 min in HV-group (*p* = 0.23) with no significant differences between the two treatment groups. Including infusion of carrier solution, the actual amount of fluid replacement between end of haemorrhage and end of dilution was 37.9 ± 4.4 ml/kg in the LV-group (=1.08 fold the amount of blood loss) and 99.7 ± 11.3 ml/kg (*p* < 0.0001) in the HV-group (= 2.85 fold the amount of blood loss) The time between blood sampling after haemorrhage and after dilution was 50 ± 19 min in LV and 57 ± 11 min in HV-group (*p* = 0.33).

After haemorrhage Hct was already significantly lower compared to baseline in both groups (Table 1). Haemorrhage alone did not decrease platelet count and WBC in both groups. Following fluid replacement, Hct, Plt and WBC significantly decreased as compared to the values after haemorrhage. Furthermore HV resulted in significant lower values of Hct and Plt and WBC as compared to LV (Table 1).

**Table 1:** Blood cell count and standard coagulation tests

	Baseline	Haemorrhage	Dilution	ANOVA
Hct (%)				
LV	29.2 ± 2.1	25.6 ± 1.4	19.9 ± 1.5	*p* < 0.0001
HV	28.3 ± 1.6	24.7 ± 1.0	16.3 ± 1.7	*p* < 0.0001
	*p* = 0.27	*p* = 0.08	*p* < 0.0001	
Plt (K/μL)				
LV	380 ± 86	399 ± 75	318 ± 67	*p* < 0.0001
HV	361 ± 62	378 ± 71	231 ± 54	*p* < 0.0001
	*p* = 0.53	*p* = 0.47	*p* = 0.002	
WBC (K/μL)				
LV	15.6 ± 3.5	16.2 ± 5.1	19.0 ± 5.5	*p* = 0.021
HV	14.4 ± 3.7	14.5 ± 3.2	12.0 ± 3.7	*p* = 0.017
	*p* = 0.43	*p* = 0.36	*p* = 0.0014	
PTI (%)				
LV	113 ± 9	110 ± 8	102 ± 7	*p* = 0.0084
HV	110 ± 6	106 ± 7	92 ± 8	*p* < 0.0001
	*p* = 0.45	*p* = 0.23	*p* = 0.0031	
aPTT (sec)				
LV	13.5 ± 0.8	14.0 ± 0.6	14.4 ± 0.6	*p* = 0.012
HV	13.5 ± 0.7	13.8 ± 0.8	14.3 ± 1.1	*p* = 0.086
	*p* = 0.85	*p* = 0.45	*p* = 0.84	
Fbg (mg/dL)				
LV	386 ± 59	268 ± 38	205 ± 19	*p* < 0.0001
HV	350 ± 67	255 ± 43	168 ± 24	*p* < 0.0001
	*p* = 0.17	*p* = 0.45	*p* = 0.0004	

Measurements at baseline, after 50 % haemorrhage and after fluid replacement (dilution) with an acetated, balanced crystalloid administering either with a low (LV, *n* = 12) or a high (HV, *n* = 12) volume strategy. *P*-values refer to differences between the two groups at each time-point. *P*-values of ANOVA refer to differences between time-points (baseline, haemorrhage, dilution)

*Hct* haematocrit, *LV* low volume resuscitation, *HV* high volume resuscitation, *Plt* platelet count, *WBC* white blood cells, *PTI* prothrombin time index, *aPTT* activated partial thromboplastin time, *Fbg* fibrinogen

Despite equal blood loss in both groups, Hct ratio between haemorrhage and dilution show a grade of blood dilution of 32 % in LV vs. 42 % in HV (*p* < 0.001). Given the actual amount of fluid administered (as compared to shed blood volume) this indicated that 59.3 % of LV fluid was still present, while only 29.5 % of HV therapy remained intravascularly at the time point of measurement. After acute blood loos of approximately 50 % blood volume, mean heart rate did not show tachycardia (90 ± 17 per min) in both groups, but frequent changes in heart rhythm and QRS deformities were observed. MAP at that time was found to be very low in both groups with no significant differences (Table 2). LV and HV crystalloid replacement stabilised MAP to 61 ± 7 mmHg in the LV group, while HV increased the blood pressure to higher levels of 82 ± 14 mmHg (*p* = 0.0002). Although heart rate did not significantly change between time points of measurement, it was significantly higher in the HV group, as compared to LV (*p* = 0.0046).

**Table 2:** Haemodynamic parameters, body temperature, and blood gas analysis

	Baseline	Haemorrhage	Dilution	ANOVA
MAP (mmHg)				
LV	107 ± 11	32 ± 10	61 ± 7	*p* < 0.0001
HV	117 ± 15	29 ± 7	82 ± 14	*p* < 0.0001
	*p* = 0.07	*p* = 0.44	*p* = 0.0002	
HR (min^−1^)				
LV	91 ± 21	86 ± 11	76 ± 17	*p* = 0.064
HV	84 ± 13	94 ± 21	90 ± 11	*p* = 0.069
	*p* = 0.32	*p* = 0.20	*p* = 0.0046	
Temperature (°C)				
LV	37.9 ± 0.5	38.0 ± 0.6	37.5 ± 0.7	*p* < 0.0001
HV	37.4 ± 0.6	37.5 ± 0.6	36.0 ± 0.6	*p* < 0.0001
	*p* = 0.046	*p* = 0.044	*p* < 0.0001	
Lactate (mg/dL)				
LV	8.5 ± 1.9	23.2 ± 8.0	17.3 ± 4.4	*p* < 0.0001
HV	8.5 ± 2.7	27.0 ± 10.3	19.7 ± 3.8	*p* < 0.0001
	*p* = 1.0	*p* = 0.32	*p* = 0.16	
pH				
LV	7.51 ± 0.03	7.48 ± 0.04	7.49 ± 0.04	*p* = 0.012
HV	7.48 ± 0.03	7.45 ± 0.04	7.50 ± 0.07	*p* = 0.041
	*p* = 0.03	*p* = 0.12	*p* = 0.67	
BE (mmol/L)				
LV	5.0 ± 1.8	1.6 ± 1.7	4.1 ± 1.7	*p* < 0.0001
HV	4.5 ± 1.3	−0.7 ± 1.7	4.4 ± 1.0	*p* < 0.0001
	*p* = 0.53	*p* = 0.004	*p* = 0.56	
HCO_3_^−^ (mmol/L)				
LV	29.2 ± 1.7	26.1 ± 1.6	28.2 ± 1.5	*p* < 0.0001
HV	28.6 ± 1.3	24.1 ± 1.4	28.6 ± 0.9	*p* < 0.0001
	*p* = 0.40	*p* = 0.004	*p* = 0.43	

Measurements at baseline, after 50 % haemorrhage and after fluid replacement (dilution) with an acetated, balanced crystalloid administering either with a low (LV, *n* = 12) or a high (HV, *n* = 12) volume strategy. *P*-values refer to differences between the two groups at each time-point. *P*-values of ANOVA refer to differences between time-points (baseline, haemorrhage, dilution)

*MAP* mean arterial pressure, *LV* low volume resuscitation, *HV* high volume resuscitation, *HR* heart rate, *BE* base excess

Baseline temperature did not decrease during haemorrhage, but through fluid replacement therapy only, as we used fluids warmed at 22 °C. HV therapy resulted in 1.5 °C decline as compared to 0.5 °C in the LV group (*p* < 0.0001, Table 2). As compared to baseline, haemorrhage resulted in significant increase in lactate and decreased pH and BE as well as bicarbonate (Table 2). As compared to baseline, PTI did not decrease with haemorrhage but with dilution in both groups, and exhibited significant lower levels in the HV-group as compared to LV (*p* = 0.0031, Table 1). aPTT did not differ between both groups after haemorrhage and dilution.

Plasma fibrinogen decreased significantly in both groups from baseline to haemorrhage (386 ± 59 mg/dL to 268 ± 38 mg/dL (LV) and 350 ± 67 mg/dL to 255 ± 43 mg/dL (HV)) with no inter-group difference (*p* = 0.45). After fluid replacement it further decreased to 205 ± 19 mg/dL in the LV group, and to 168 ± 24 mg/dL in the HV group (*p* = 0.0004).

**Fig. 2:**
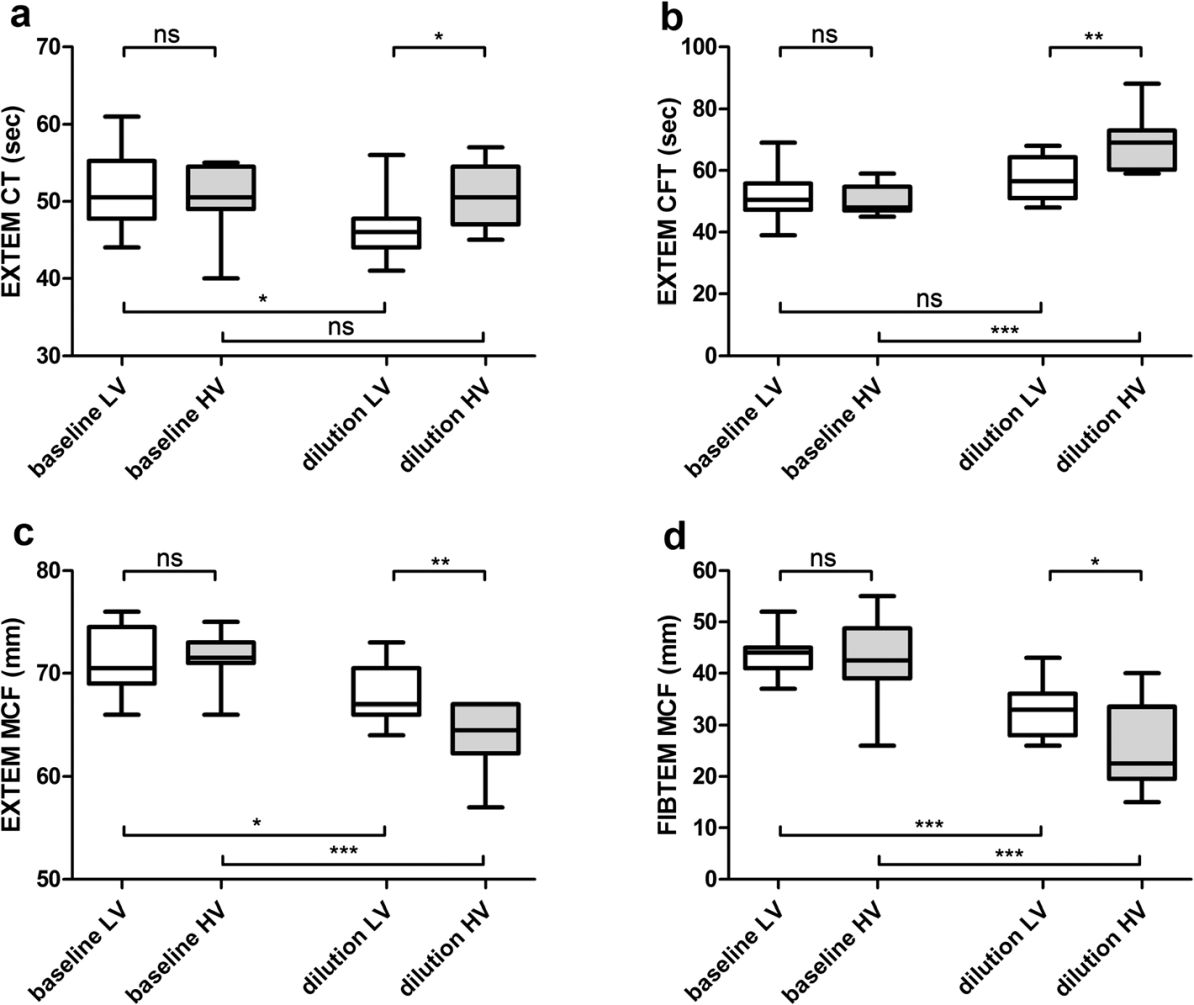
Thromboelastometric measurement of whole blood, using EXTEM (**a**) clotting time (CT), (**b**) clot formation time (CFT), and (**c**) maximum clot firmness (MCF) as well as (**d**) FIBTEM MCF. Data presented are median, interquartile range and range. *Shaded boxes* represent the high volume (HV) group; *white boxes* represent the low volumen (LV) group. *ns* not significant

ROTEM results in whole blood, using the EXTEM assay (CT, CFT, MCF) as well as the FIBTEM assay (MCF) are presented in Fig. 2a-d. In the LV group, haemorrhage and dilution resulted in a shortening of CT as compared to baseline, whereas it did not change in the HV group. CFT did not change in the LV group, but was significantly prolonged in the HV-group. MCF of the EXTEM as well as the FIBTEM assay was significantly reduced in both groups as compared to baseline. Thromboelastometric clot strength of extrinsic activated platelet free plasma decreased in response to haemodilution from baseline MCF (24 ± 2 mm in LV, versus 24 ± 3 in HV, *p* = 0.87) to (16 ± 2 versus 13 ± 2 mm, respectively. Clot strength was significantly impaired in the HV-group (*p* = 0.0039).

For all ROTEM parameters it was observed, that HV significantly weakened viscoelastic coagulation properties more than did LV fluid replacement. With exception of a significant decrease in potassium after HV-therapy as compared to LV, no differences of electrolyte and glucose levels were observed between the two groups of fluid replacement (Table 3).

**Table 3:** Electrolytes and glucose

	Baseline	Haemorrhage	Dilution	ANOVA
Sodium (mmol/L)				
LV	140 ± 3	139 ± 2	139 ± 3	*p* = 0.037
HV	138 ± 2	138 ± 1	139 ± 1	*p* = 0.033
	*p* = 0.10	*p* = 0.11	*p* = 0.43	
Potassium (mmol/L)				
LV	3.7 ± 0.4	4.1 ± 0.3	3.7 ± 0.1	*p* = 0.01
HV	3.6 ± 0.2	4.0 ± 0.4	3.4 ± 0.2	*p* < 0.0001
	*p* = 0.31	*p* = 0.48	*p* < 0.0001	
Cloride (mmol/L)				
LV	104 ± 3	105 ± 2	105 ± 3	*p* = 0.31
HV	106 ± 2	108 ± 1	107 ± 1	*p* < 0.0001
	*p* = 0.14	*p* = 0.0003	*p* = 0.067	
Calcium (mmol/L)				
LV	1.32 ± 0.08	1.33 ± 0.08	1.27 ± 0.05	*p* = 0.17
HV	1.30 ± 0.07	1.29 ± 0.06	1.28 ± 0.06	*p* = 0.67
	*p* = 0.57	*p* = 0.18	*p* = 0.70	
Glucose (mg/dL)				
LV	111 ± 18	120 ± 19	91 ± 18	*p* < 0.0001
HV	110 ± 21	146 ± 43	103 ± 17	*p* = 0.0019
	*p* = 0.92	*p* = 0.062	*p* = 0.12	

Measurements at baseline, after 50 % haemorrhage and after fluid replacement (dilution) with an acetated, balanced crystalloid administering either with a low (LV, *n* = 12) or a high (HV, *n* = 12) volume strategy

*LV* low volume resuscitation, *HV* high volume resuscitation

## Discussion

In this preclinical in vivo study, using a specific haemodilution model with controlled bleeding prior to fluid administration, we could show that a LV regimen of the investigated balanced crystalloid solution resulted in a higher percentage of intravascular volume expansion of 60 % as compared to 30 % in HV within the first hour of application with regard to haematocrit. Furthermore it was observed that even a high volume of this balanced crystalloid did not result in hyperchloraemic acidosis. We could also show that in the acute setting of 50 % controlled blood loss, a LV crystalloid replacement strategy is sufficient to adequately raise blood pressure up to a mean arterial pressure >50 mmHg .[22]. In contrast to HV replacement strategy that results in higher blood pressure, the concept of damage control resuscitation (DCR) with permissive hypotension may be better met by using LV.

In addition to the favourable effect of lower MAP in the LV group, coagulation parameters represented by plasma fibrinogen and ROTEM parameters are also less impaired with LV. Nevertheless, hypoperfusion in combination with low blood pressure might cause lower tissue oxygenation that may also reduce plasma fibrinogen levels [23, 24]. Time of withdrawl (i.e. haemorrhage) presumably has an impact on shock and haemostasis, but as time was comparable in the LV and HV group (*p* = 0.23), there was no substantial effect on the results of the study.

There was no significant change in heart rate, as pigs were anaesthetised with midazolam and sufentanil, with the latter substance to be known to blunt cardiac chronotropic response to hypotension.

### Use of crystalloid for volume replacement

Crystalloid fluids are widely used for volume resuscitation. The “simplest” crystalloid, normal saline, is a solution of 0.9 % of sodium chloride, with an osmolality of 308 mosmol/L. It contains 154 mmol/L of sodium and 154 mmol/L of chloride and therefore this solution can be considered neither physiological nor balanced. However, saline is frequently used as a plasma substitute [25]. In contrast, balanced electrolyte solutions are isotonic and have electrolyte compositions close to that of plasma [26]. Although there are still few studies where outcome benefit has been shown [27], there is little reason to question the rationale for using balanced solutions [5, 28].

The crystalloid solution used in the current study offers balanced electrolyte content with chloride of 108 mmol/L, but using a high content of acetate, reducing the probability of inducing hyperchloraemic acidosis. Manufacturer’s recommendations suggest a maximum daily dose of 30–40 ml/kg. Considering lactate as a surrogate parameter for tissue oxygenation, it was shown that crystalloid fluid administration was able to significantly improve lactate clearance as compared to haemorrhage. Importantly, after fluid replacement, neither lactate, pH, nor BE differed between both groups.

### Volume effect of the balanced crystalloid

The intravascular volume expansion effect of crystalloid fluids is low [29]. Therefore, large amounts of crystalloids are often necessary to provide sufficient increase in intravascular volume. Following infusion of crystalloid solutions, 80 % and more cross the capillary membrane from the intravascular compartment into the interstitial space within minutes [12]. Our results showed that after a blood loss of 35 ml/kg (50 % of assumed blood volume of 70 ml/kg) administration of an equal amount of crystalloid solution (LV-group) did cause a dilution effect of 32 % of the remaining blood, suggesting that approximately 60 % of administered crystalloid volume was still intravascular within the first hour of administration. In HV, the grade of blood dilution was found to be 42 % indicating that only about 30 % of the actually administered fluid volume remained intravascular at the same time point of measurement. The latter is in accordance to further studies showing a time dependent 20–30 % volume effect of crystalloids [14, 15]. In the current specific setting of massive but controlled blood loss, administration of low volumes of crystalloid exerts a preferential acute volume effect. Whether this could be attributed to the actual need of the exsanguinating animal or patient needs to be confirmed.

Mean arterial blood pressure in the LV-group after volume replacement was 61 ± 7 mmHg, thereby rose to a clinically acceptable level (>50 mmHg) [22], when considering blood pressure as a commonly used global perioperative clinical surrogate parameter for perfusion in bleeding patients. In contrast, HV raised MAP to 82 ± 14 mmHg, suggesting overtreatment, with regard to the concept of DCR with deliberate permissive hypotension [22, 30]. We are, however, aware that we did not investigate an uncontrolled bleeding model, limiting interpretation of these specific results with regard to DCR. Nevertheless, as the animal was exposed to 30 min of bleeding with severe blood loss of 50 %, we imitated clinical reality of “uncontrolled bleeding” until surgical supply. Fluid resuscitation, administered at room temperature, caused body temperature to significantly fall, with the HV strategy even more than did LV. This means that fluid administration in general may enhance an important negative effect–via lower temperature–on coagulation. On the other side fluid resuscitation may improve tissue oxygenation, thus resulting in less acidosis, another factor influencing coagulation. Our study could show that the LV strategy is preferential with regard to the combination of these two parameters.

### Impact on coagulation

HV decreased PTI significantly more than LV. Interestingly, aPTT was not different between both groups. This is in accordance with an in vitro model of dilutional coagulopathy where it was shown that aPTT is less affected by dilution than PTI [31]. The influence on plasma fibrinogen, considered to be the first coagulation factor to fall below critical levels [21], was different between both treatment regimens with preferential effects of LV. In both groups fibrinogen decreased during the phase of haemorrhage, which might be due to consumption of coagulation factors in the preparation phase, dilution by shift of intracellular and interstitial fluids into the vascular space [32], and increasing hypoxia due to worsened tissue oxygenation [23, 24]. It has been shown that fibrinogen plays a crucial role in primary and secondary haemostasis. Hypofibrinogenaemia is strongly related to the severity of shock and the amount of blood loss, and fibrinogen is almost always the coagulation factor which reaches critical low levels first [33]. This fact is rather a result of a combination of hypoperfusion and consecutive tissue hypoxemia as well as acidaemia than the effect of one single trigger. Sour environmental conditions following hypoperfusion lead to increased breakdown of fibrinogen and might have additionally negatively affected utilisation of fibrinogen with high levels of soluble thrombomodulin and Prot C [34, 35]. Resuscitation and consecutive dilution are aggravating factors of hypofibrinogenaemia, and dilution according to different fluid resuscitation regimen leads to further reduction of fibrinogen levels.

Haemorrhage inevitably results in adverse outcome, elsewise aggressive reconstitution of coagulation components and circulating volume is timely initiated. Damage control resuscitation in case of acute trauma haemorrhage with blood components only does not consistently improve coagulation potential [36]. Moreover, transfusion of red blood cells might further “dilute” coagulation factors, and only substitution of components with a total high fibrinogen load substantially enhance coagulation [37].

Lower platelets and lower plasma fibrinogen in the HV group may also explain the significant higher impact of HV on viscoelastic clot properties. In the present study, prolongation of CFT and reduction of MCF suggested weakened coagulation capacity after haemorrhage and fluid replaced, with a significant impact when using HV. However, it is of interest that in vivo haemodilution of approximately 32 % (i.e. LV) shortened CT. This is in line with a previous in vitro observation, which showed the same effect of a 33 % haemodilution using saline [38]. Shortened CT suggests enhanced thrombin generation. Dunbar et al. showed increased thrombin generation following dilution of plasma proteins [39]. A study by Ruttmann and colleagues confirmed a faster onset of coagulation, an increased rate of clot formation and an increase in cloth strength after the initial fluid load in the setting of vascular surgery. The observed shortening of the thromboelastographic r-time, corresponding to the thromboelastometric CT in our study, could be interpreted as an imbalance between activated pro-coagulants and a reduction in anticoagulants, particularly AT III [40].

Another explanation is that in case of traumatic injury procoagulant particles of different origin are being released into circulation that lead to enhanced thrombin generation in trauma patients. These microparticles are mainly released from activated cells like endothelial cells, platelets, but also from erythrocytes and immunological cells. Moreover, circulating particles directly related to the site of injury like collagen, tissue factor, apoptotic cell parts or phospholipids might contribute to increased thrombin generation [41, 42].

### Impact on electrolytes and glucose

No differences of electrolyte and glucose levels were observed between the two forms of fluid replacement, with one exception, namely potassium. Despite the fact that ELO-MEL contains 5 mmol/L of potassium, the HV strategy resulted in lower potassium values than did LV. The reasons for this may lay in the capability of cells to exchange potassium for protons.

High volumes of unbalanced solutions may lead to hyperchloraemic acidosis. The administration of the currently investigated modern acetated balanced crystalloid did not result in hyperchloraemic acidosis, even at high volumes of 100 ml/kg. This may furthermore support the use of balanced crystalloids in bleeding patients [5, 28].

### Limitations

Though various swine models of haemorrhage are used for studies on resuscitation and coagulation [16–18], we have to take into consideration that we cannot directly transfer the results to human clinical reality. In comparison to human individuals pigs present in a hypercoagulabe state.

As thromboelastometric measurements have not been performed at the “haemorrhage point”, we cannot rule out affection of the resuscitation data due to possible induction of a hypocoagulable condition according to shock.

In this study measure points were set in a narrow time frame to evaluate LV versus HV resuscitation, so we cannot draw any conclusions on medium-term or long-term effects of each resuscitation regimen. Nevertheless, initial fluid resuscitation with LV of crystalloids proves to be sufficient in our setting in the acute phase, and might prevent from the potential adverse effects of high volume fluid resuscitation on shedding of glycocalyx and haemostasis [43].

## Conclusion

In a pre-clinical in vivo model with 50 % controlled blood loss, a high volume administration (three-fold shed blood volume) of an acetated balanced crystalloid does not lead to hyperchloraemic acidosis. It may, however, unfavorably influence clinical parameters, such as higher blood pressure, lower body temperature and impaired coagulation, which could potentially increase bleeding tendency in trauma patients. With respect to the fact that we conducted our study in a swine model of haemorrhage, replacement of acute blood loss with just an equal amount of an acetated balanced crystalloid appears to be the preferential treatment strategy in the acute phase after controlled bleeding in this animal model. Clinical trials regarding appropriate crystalloid fluid replacement after severe haemorrhage are necessary, but presumably hard to establish.

## Abbreviations

LV: Low volume group
HV: High volume group
MAP: Mean arterial pressure
PTI: Prothrombin time index
aPTT: Activated partial thromboplastin time
Hct: Haematocrit
Plt: Platelet count
WBC: White blood cell
PFP: Platelet free plasma
CT: Clotting time
CFT: Clot formation time
MCF: Maximum clot formation
BE: Base excess
DCR: Damage control resuscitation
